# Cyclopædia of the Diseases of Children

**Published:** 1890-12

**Authors:** 


### Book Reviews.

---

CYCLOPÆDIA OF THE DISEASES OF CHILDREN. Edited by John M. Keating, M. D. Published by J. B. Lippincott Company, Philadelphia.

We have received volumes 1 and 2 of this work, and by the merit of these judging those which are to follow, we are convinced that it will be one of the most complete and comprehensive systems of medicine ever offered the profession.

The volumes are bound in cloth or sheepskin, and the body is formed of the most durable and elegant paper. The subject matter consists of treatieses from the pens of the ablest and most experienced writers of Europe, Canada and America, who have spared no pains to furnish their readers with the recent and reliable pathological and therapeutical discoveries in this department, all of which is set forth in legible type and simplified by numerous illustrative plates. The matter is so arranged as to render it alike suited to the busy practitioner as a ready reference, and for the student in further search for detailed information.

Part 1 of volume 1 is devoted to general subjects, e. g., the anatomy and physiology of infancy and childhood, diagnosis, outline of practical bacteriology, maternal impression and infant feeding.

Part II. gives a full discussion of fevers and miasmatic diseases, while the chapter on monstrosities affords valuable and interesting information to those seeking knowledge in this direction. This volume closes with a practical treatise on general therapeutics of children's diseases.

Volume 2d begins with diseases of the skin, and embodies constitutional diseases, diseases of the respiratory tract, diseases of the throat and glandular system, and diseases of the buccal cavity.

We are attracted by the particular attention given to details, the chief essential to success in the diagnosis, management and treatment of diseases peculiar to this period of life.

The ready acceptance with which this work has met bespeaks the high esteem in which it is already held, and sustains us in commending it to those desirous of a valuable and useful addition to their store of medical liturature.

